# The Iowa and Illinois Central District Medical Association

**Published:** 1867-03

**Authors:** 


### THE IOWA AND ILLINOIS CENTRAL DISTRICT MEDICAL ASSOCIATION.

On the 9th of January, the following named society sprung into existence, at Davenport, Iowa:—"The Iowa and Illinois Central District Medical Association." The following officers and committees were elected and appointed. The committees are to report at the April meeting, to be held in Rock Island:

*For President*—Dr. Patrick Gregg, Rock Island.

*For Vice-President*—Dr. P. J. Farnsworth, Clinton, Iowa.

*For Treasurer*—T. J. Iles, Davenport, Iowa.

*For Secretary*—W. F. Peck, Davenport, Iowa.

*Committee on Prevailing Diseases*—Dr. Charles Plummer, Rock Island, Chairman. Drs. P. J. Farnsworth, Clinton, Iowa; Jas. McNeil, DeWitt, Iowa; Jas. Gamble, LeClaire, Iowa; James Witherwax, Davenport, Iowa. The Chairman was instructed, by resolution, to communicate with the city councils in this section of country, advising them to adopt measures by which epidemic diseases may be checked in their progress.

*Committee on Obstetrics*—Dr. Truesdale, Chairman, Rock Island. Drs. Vincent, Hampton, Ill.; Herford, Davenport, Iowa.

*Committee on Surgery*—Dr. W. F. Peck, Chairman, Davenport, Iowa. Drs Miller, Bellevue, Iowa; Widerman, Rock Island.

*Committee on Medicine*—Dr. Richardson, Chairman, Davenport. Drs. Tomson, Davenport; Graham, Bellevue, Iowa.

*Committee on New Remedies*—Dr. A. S. Maxwell, Chairman, Davenport. Drs. Brybee, Rock Island; Irwin, Davenport.

*Committee on Phthisis Pulmonalis*—Dr. Jno. Bell, Chairman, Davenport. Drs. Baker, Davenport; Lyford, Port Byron, Ill.

Dr. P. Gregg, of Rock Island, President elect, addressed the Association at some length, in language and matter peculiarly appropriate to the new organization.

Dr. Plummer, of Rock Island, read a very interesting paper on cholera, which elicited valuable discussion.

Dr. Peck will read a paper at the next meeting, on pelvic cellulitis.

The next meeting will be held in Rock Island, on the second Wednesday in April.

